# Isolation and Characterization of *Bacillus cereus* Phage vB_BceP-DLc1 Reveals the Largest Member of the Φ29-Like Phages

**DOI:** 10.3390/microorganisms8111750

**Published:** 2020-11-07

**Authors:** Chun Li, Xiaoming Yuan, Na Li, Juan Wang, Shubo Yu, Haiyan Zeng, Jumei Zhang, Qingping Wu, Yu Ding

**Affiliations:** 1 Department of Food Science and Technology, Institute of Food Safety and Nutrition, College of Science & Engineering, Jinan University, Guangzhou 510632, China; lic18@jnu.edu.cn (C.L.); yxm0324@stu2018.jnu.edu.cn (X.Y.); ln1997@stu2019.jnu.edu.cn (N.L.); 2 College of Life Science and Technology, Jinan University, Guangzhou 510632, China; 3 State Key Laboratory of Applied Microbiology Southern China, Guangdong Institute of Microbiology, Guangdong Academy of Sciences, Guangzhou 510070, China; dingyu@gdim.cn (S.Y.); zenghy@gdim.cn (H.Z.); zhangjm@gdim.cn (J.Z.); wuqp@gdim.cn (Q.W.); 4 Guangdong Provincial Key Laboratory of Microbial Safety and Health, Guangdong Institute of Microbiology, Guangdong Academy of Sciences, Guangzhou 510070, China; 5 Guangdong Open Laboratory of Applied Microbiology, Guangdong Institute of Microbiology, Guangdong Academy of Sciences, Guangzhou 510070, China; 6 College of Food Science, South China Agricultural University, Guangzhou 510642, China; wangjuan@scau.edu.cn

**Keywords:** *Bacillus cereus*, bacteriophage, φ29, stability, adsorption, largest genome

## Abstract

*Bacillus* phage φ29 and its relatives have been considered as one of the most important model organisms for DNA replication, transcription, morphogenesis, DNA packaging studies, and nanotechnology applications. Here, we isolated and characterized a new member of the φ29-like phage, named *Bacillus cereus* phage vB_BceP-DLc1. This phage, with a unique inserted gene cluster, has the largest genome among known φ29-like phages. DLc1 can use the surface carbohydrate structures of the host cell as receptors and only infects the most related *B. cereus* strains, showing high host-specificity. The adsorption rate constant and life cycle of DLc1 under experimental conditions were also determined. Not only stable under temperatures below 55 °C and pH range from 5 to 11, the new phage also showed tolerance to high concentrations of NaCl, 75% ethanol, chloroform, and mechanical vortex, which is preferable for practical use in the food and pharmaceutical industries.

## 1. Introduction

*Bacillus cereus* is a Gram-positive, spore-forming, opportunistic pathogen widely spread in the environment and may provoke harmful foodborne illness [1] as well as other infections, such as wound infections, bloodstream infection, umbilical cord infection in neonates, and respiratory tract infections, etc. [2,3]. Antibiotic treatment is still the primary means to eradicate pathogenic *B. cereus* if this bacterium causes serious infections, but the rapid expansion of antibiotic resistance of *B. cereus* has led to growing difficulty in practical treatment and requires the antibiotic use to be strictly controlled [4,5]. Thus, it is urgent to develop alternative methods to control pathogenic *B. cereus* strains in advance.

As the most abundant and diverse biological entities on our planet, bacteriophages (aka phages) can be found in every explored biome [6]. Phages are natural predators of bacteria and were applied clinically as early as the 1920s [7]. With the rapid development of antibiotic resistance, phage therapy has been revitalized as an alternative for antibiotics [8]. Phages are much more specific than antibiotics, minimizing the impact on commensal bacteria essential for humans [9]. On the other hand, phages can coevolve with their hosts, dealing with the mutation of bacteria [10]. Furthermore, different phages can also be used together as phage cocktails [11] or combined with antibiotics [12] to conquer the development of phage resistance and drug tolerance.

Plenty of phages preying on *B. cereus* strains have been discovered, all of which are grouped into the families of *Myoviridae*, *Siphoviridae*, and *Tectiviridae* phages, whereas little is known about the *Podoviridae* phages infecting *B. cereus*, as reported in 2014 [13]. As far as we know, only a few phages (VMY22, DK1, DK2, and DK3) isolated from *B. cereus* could be categorized into the *Podoviridae* family until now [14,15]. If we further look into the *Bacillus cereus* sensu lato group, which contains *B. anthracis*, *B. cereus* sensu stricto (*B. cereus*), *B. thuringiensis*, *B. weihenstephanensis*, and many other species [16,17], more *Podoviridae* phages can be identified, including QCM11 in *B. anthracis* [18], MG-B1 in *B. weihenstephanensis* [19], and some other phages, represented by Goe4, using *B. thuringiensis* as the host [20]. Notably, all of these *Podoviridae* phages have high similarity with the *B. subtilis* phage φ29 in their morphologies and genome components, so could be integrated into the φ29-like group [20]. There are several common characteristics in phages of the φ29-like group. Firstly, they are lytic phages and harbor small linear double-stranded DNA with a phage-encoded terminal protein (TP) covalently linked to each 5′ end, which participates in a special protein-primed DNA replication together with a high-fidelity DNA polymerase [21]. Secondly, a short inverted terminal repeat (ITR) region is conserved in the genome termini of φ29-like phages, with a reiteration of at least two nucleotides at the 3′ ends which is required in a sliding-back mechanism and ensures that the initiation of replication occurs with high fidelity [22]. The third unique feature is the molecular motor exploited by the φ29-like phages to pack DNA into their capsids, which is composed of a connector (upper collar protein), an ATPase (DNA encapsidation), and a pro-head RNA (pRNA) acting as a linker element, and is regarded as one of the most powerful mechanical devices ever characterized [23]. Phages in the φ29-like group are an important repository for novel enzymes in molecular biology and can also provide insights into evolution mechanisms between phages and their host [20].

Currently, as officially approved by the International Committee on Taxonomy of Viruses (ICTV) [24], the φ29-like phages are categorized into the *Salasvirus* genus of the *Picovirinae* subfamily, which contains only three historical species, φ29, B103, and GA1, using *B. subtilis* as the host. Conversely, other φ29-like phages isolated from the *Bacillus cereus* sensu lato group, especially *B. cereus* phages, are poorly characterized and temporarily not classified in detail. Considering that the φ29-like phages are the only known *Podoviridae* phages infecting the *Bacillus cereus* sensu lato group and the importance of φ29-like phages in molecular biology, the integration of taxonomic information about these phages is imperative. Moreover, for the sake of practical application in controlling pathogenic *B. cereus*, it is necessary to characterize lytic phages not only from gene level but also in their availability in various environments, especially for some unique phages. Therefore, in this study, we identified a novel φ29-like *B. cereus* phage, vB_BceP-DLc1. Its morphology and genomic features were characterized, and its position in taxonomy was proposed based on the comparison of related phages. Furthermore, its stability under different environmental stresses, as well as its life cycle and adsorption capacity toward bacteria, were also evaluated. This newly isolated phage here can broaden our knowledge of the φ29-like group and potentially be applied as a promising candidate for phage cocktails in the prevention and control of *B. cereus*.

## 2. Materials and Methods

### 2.1. Bacterial Strains and Growth Conditions

Tryptic soy broth (TSB) (Huankai, Guangzhou, China) and agar (Huankai) were used for propagation. *Bacillus cereus* strains were obtained from the Guangdong Institute of Microbiology and stored at −40 °C in 25% (*v*/*v*) glycerol.

### 2.2. Isolation and Purification of DLc1

The phage vB_BceP-DLc1 was isolated from the sewage samples collected in the Huangsha aquatic product market (Guangzhou, China) using *Bacillus cereus* 1582-3B as the host. The sewage samples were pre-treated by the adsorption-elution method described earlier [25] to remove environmental bacteria and obtain the concentrated environmental virus mixture. To enrich the specific phage, this virus mixture (300 μL) was co-cultured with the host strain 1582-3B (30 μL) in 3 mL TSB supplemented with 2 mM CaCl_2_ (Shanghai Yuanye Bio-Technology Co., Ltd., Shanghai, China) (shaking at 200 rpm, 37 °C for 1.5 h, and standing steadily at 37 °C for 24 h), and the raw DLc1 phage suspension was obtained after centrifugation (10,000 × *g* at 25 °C for 1 min) and filtration (0.22-μm sterile syringe filter). DLc1 phage was purified as follows [26]: streaking the raw suspension onto TSB base plate (1.5% *w*/*v* agar; containing 1 mM CaCl_2_) using 1 μL inoculating loop and air-dried; carefully pouring 4 mL TSB soft agar (0.4% *w*/*v* agar; containing 1 mM CaCl_2_) inoculated with 100 μL exponential host cells from the most diluted area; slightly tilting the plate and allowing the soft agar to cover the full area. After solidification, the plate was incubated at 37 °C overnight. The well-separated single plaque was picked up and the purification process was repeated at least three times.

### 2.3. Preparation of DLc1 Phage Stock with High Titer

The DLc1 stock with a high titer was prepared by three-step amplification and polyethylene glycol (PEG) precipitation. A purified single plaque was first picked up and dispersed into 3 mL TSB (containing 1 mM CaCl_2_) medium, which was inoculated with 30 μL overnight host cells and cultured for 3 h (37 °C, 200 rpm). The 1st-step supernatant was obtained after centrifugation (10,000× *g* at 37 °C for 1 min) and filtration through a 0.22-μm syringe filter. Thereafter, another 3 mL TSB (containing 1 mM CaCl_2_) was inoculated with 30 μL overnight host cells and allowed to grow for 1 h (37 °C, 200 rpm) before adding 100 μL of 1st-step supernatant. Then, the mixture was shaken for another 6 h under the same conditions and the 2nd-step supernatant was separated the same as above. Finally, 50 mL of TSB (containing 1 mM CaCl_2_) was inoculated with 500 μL overnight host cells and allowed to grow for 1 h (37 °C, 200 rpm) before adding 1 mL of 2nd-step supernatant. Then, the mixture was shaken overnight under the same conditions. The 3rd-step supernatant was separated after centrifugation (10,000 × *g* at 4 °C for 20 min) and filtration through a 0.22-μm sterile syringe filter. Condensation of the phages was conducted by adding 10% PEG (MW = 8000; BBI Life Sciences, Shanghai, China) and 1 M NaCl (Shanghai Titan Scientific Co., Ltd., Shanghai, China), and then the phage particles in the 3rd-step supernatant were precipitated in an ice-bath for 4 h. After centrifugation (10,000× *g* at 4 °C for 30 min), the precipitates were re-suspended in 2 mL of ultrapure water (Type 1; Millipore, Shanghai, China) and stored at 4 °C with the titer of ~ 2.5 × 10^12^ PFU/mL.

### 2.4. Transmission Electron Microscopy

Samples were deposited on carbon-coated copper grids and negatively stained with 3% phosphotungstic acid for 3 min. Morphology and adsorption behavior of DLc1 were visualized by transmission electron microscopy (TEM; Hitachi H-7650, Tokyo, Japan) at an acceleration voltage of 80 kV with a CCD camera. Average size of DLc1 was obtained from at least 20 individual virions and measured by ImageJ software (version 1.52d, National Institutes of Health, USA). For the observation of adsorption behavior, *B. cereus* 1582-3B, treated with sodium periodate (10 mM) or proteinase K as described in adsorption assays, was infected with DLc1 at a multiplicity of infection (MOI_added_) of 100 (10^10^ PFU/mL to 10^8^ CFU/mL) and subjected to TEM after 10 min adsorption at 37 °C.

### 2.5. Genome Sequencing and Analysis

Genomic DNA of DLc1 was extracted and purified by the phenol-chloroform method [27]. Briefly, phage suspension was successively incubated with ribonuclease A (RNase A; 50 μg/mL, Takara, Beijing, China) and recombinant DNase I (42 U/μL, Takara) in a 1.5-mL Eppendorf tube at 37 °C for 1 h to remove bacterial RNA and DNA. The sample was subsequently treated with sodium dodecyl sulfate (SDS; 0.3%), ethylenediamine tetraacetic acid disodium salt (EDTA; 17 mM), and protease K (42 μg/mL) at 65 °C for 1 h to digest the phage capsid. An equal volume of phenol was added and the mixture was centrifuged (12,000 × *g* at 4 °C for 5 min) after vigorous vortex for 30 s. The top aqueous layer was transferred to a new tube and an equal volume of a phenol-chloroform-isoamylol mixture (25:24:1; *v*:*v*:*v*) was added and the tube was centrifuged (12,000 × *g* at 4 °C for 5 min) after vigorous vortex for 30 s. The aqueous layer was washed three times with chloroform and mixed with an equal volume of isopropanol and stored at −20 °C for 30 min. After centrifugation at 12,000 × *g* at 4 °C for 20 min, the DNA pellet was washed twice with 75% ethanol solution and finally re-suspended with sterile ultrapure water.

The phage genome was sequenced by the Ion torrent S5 platform (Thermo Fisher Scientific, Waltham, MA, USA). The DNA library was prepared and the sequencing was performed following the manufacturer’s instructions [25]. The initial assembly was performed with SPAdes (version 3.12.0) [28]. Genome ends were verified by Sanger sequencing (BGI, Shenzhen, China) using the primers 190926_R (5′ AGATCTGATTATTGCGTGTCC) and 190926_L (5′ GATATTCCACGGTGAAACGTGCC). The average nucleotide identity (ANI) was calculated using MUMmer (ANIm) alignment method in the pyani package [20] and the Hadamard matrix was plotted in an interactive heatmap using R [29,30]. The host strain, DNA scale, GC content, current taxonomy, and accession number of each phage used for alignment are listed in Table S1. Prokka 1.13.7 [31] was used for the open reading frame (ORF) detection and initial genome annotation. Further functional annotation was manually completed by BLASTp against the non-redundant protein database in NCBI and InterProScan [32]. The pro-head RNA (pRNA) in the genome of DLc1 and other phages was identified by the Infernal 1.1.3 software package [33] (Table S2). The required co-variance model was calculated using the pRNA gene sequences from virus Nf (NC_049976) and GA-1 (NC_002649) as input CM file, which was provided by Schilling et al. [20]. Pairwise genome comparison was done by tblastx [34] and visualized using Easyfig 2.2.3. [35]. The similarity of protein pairs was calculated with Needleman–Wunsch algorithm using the needle program of EMBOSS suite [36] with the default parameters.

### 2.6. Phylogenetic Analysis

The phylogenetic analysis was conducted by MEGA X [37]. In phages from the *Picovirinae* subfamily, genes coding four typical proteins, including DNA polymerase, DNA terminal protein, pre-neck appendage protein, and endolysin, were used as markers. The available DNA sequences in NCBI coding proteins were aligned by MUSCLE [38] using default parameters. Gaps were removed before alignment. The phylogeny was reconstructed using the neighbor-joining method based on the p-distance substitution model [39] and tested by the bootstrap method with 1000 replications.

### 2.7. Stability of DLc1

To determine the temperature stability of DLc1, phage was suspended in ultrapure water or TSB, respectively, to a final titer of 1 × 10^8^ PFU/mL (pH 7.0). The phages were kept in a refrigerator (4 °C), an incubator (25 °C), or subjected to a water bath at different temperatures (37, 45, 55, 65, and 75 °C), respectively, for 1 h.

For the pH stability test, the pH value of ultrapure water was adjusted to 1, 3, 5, 7, 9, 11, and 13, respectively, using 0.1 M HCl and 0.1 M NaOH solution. The phage was suspended in the above solution to a final titer of 1 × 10^8^ PFU/mL and kept in dark at 25 °C for 1 h.

Stability of DLc1 in NaCl and ethanol solution was measured by suspending phage in NaCl solution with different concentrations (50, 100, 200, 300, 400, 500, and 1000 mM) and different ethanol solutions (10, 25, 50, 75, and 90%) to a final titer of 1 × 10^8^ PFU/mL and kept at 4 °C for 1 h.

To test the sensibility of DLc1 to chloroform and mechanical vortex, 500 μL phage suspended in ultrapure water (1 × 10^8^ PFU/mL) was mixed with the same volume of chloroform (Sinopharm Chemical Reagent Co., Ltd., Shanghai, China). The tube was then vortexed for different time periods (5, 30, 60, and 120 s) and left to stand for 15 min on ice before titer determination. An additional tube was also vortexed for 5 s and left to stand for 24 h at 4 °C. The phage suspensions (1 mL) without adding chloroform were treated simultaneously under the same conditions. The titer before treatment was set as the control.

The titer in the stability experiments was determined by small drop plaque assay [40], using *B. cereus* 1582-3B as the indicator strain. All experiments were performed in triplicate and the results were represented as the mean count with standard deviations.

### 2.8. Host Range Determination

The host range of DLc1 was determined by the spot assay. DLc1 phage (1 × 10^8^ PFU/mL) was 10-fold serially diluted and 5 μL of each dilution was spotted onto the TSB soft agar overlay containing the tested strains grown exponentially. The plate was incubated at 37 °C for 4 to 12 h and the bacterium showing plaque formation was identified as the potential host of DLc1.

### 2.9. Adsorption Rate Constant k

The adsorption rate constant *k* of DLc1 was determined following the recommendations of Hyman and Abedon [41]. A portion of exponentially growing *B. cereus* strain 1582-3B (1.5 mL; OD_600_ 2.0; 1 × 10^8^ CFU/mL) was harvested (13,000 × *g* at 25 °C for 1 min) and re-suspended in 1.5 mL TSB (containing 1 mM CaCl_2_). The suspension was diluted 10 times with the same medium (1 × 10^7^ CFU/mL) and supplemented with 25 μg/mL of chloramphenicol (Shanghai Titan Scientific Co., Ltd., Shanghai, China) to inhibit cell growth and phage multiplication [42]. After dilution with TSB (containing 1mM CaCl_2_), phage and cell suspensions were both pre-warmed at 37 °C for 5 min and mixed at a MOI_added_ of 0.001 (1 × 10^4^ PFU/mL). The volume was set as 10 mL and the adsorption was conducted in a 50-mL round bottom polypropylene tube at 37 °C for 10 min. Samples (500 μL) were taken every minute using 1-mL syringe and filtrated through a 0.22-μm syringe filter, and the un-adsorbed phage was enumerated by double agar overlay plaque assays [43]. A bacteria-free and phage-free setup served as the negative control, and the concentration of bacterial cells was enumerated by plate count method [44]. The experiments were performed independently in triplicate and the adsorption rate constant *k* of each experiment was calculated as follows [45]:

(1) $$k=\frac{-s}{N}$$

where *k* is the adsorption rate constant, N is the concentration of bacterial cells, and s is the slope of the linear regression curve of ln (P_t_/P_0_) plotted against time (t), in which P_t_ is the un-adsorbed phage counted at time t and P_0_ is the phage counted at the beginning.

### 2.10. One-Step Growth Curves

A portion of exponentially growing *B. cereus* strain 1582-3B (1.5 mL; OD_600_ 3.0; 2 × 10^8^ CFU/mL) was harvested (13,000 × *g* at 25 °C for 1 min) and re-suspended in 1.5 mL TSB (containing 1 mM CaCl_2_). The suspension was diluted 10 times with the same medium (2 × 10^7^ CFU/mL). Phage and cell suspensions were both pre-warmed at 37 °C for 5 min and mixed at a MOI_added_ of 0.1 (2 × 10^6^ PFU/mL). After adsorption for 5 min at 37 °C, 50 μL samples were 1000-fold diluted into 50 mL TSB (containing 1 mM CaCl_2_) to prevent subsequent phage adsorption [41]. The mixture was incubated at 37 °C with shaking (120 rpm) and samples (500 μL) were withdrawn every 5 min and filtrated through a 0.22-μm sterile syringe filter. To determine the eclipse period of DLc1, the second series of samples (500 μL) was also removed every 5 min and mixed with 1% chloroform to release intracellular phages and filtrated through a 0.22-μm syringe filter. Phages in the mixture were quantitated by the double agar overlay plaque assay. The experiments were carried out independently in triplicate and the one-step growth curve was plotted based on the mean values. The latent and eclipse period of DLc1 was directly read and the burst size of DLc1 was calculated as follows:

(2) $$\mathrm{Burst}\;\mathrm{size}=\frac{\mathrm{Phage}\;\mathrm{amount}\;\mathrm{after}\;\mathrm{burst}-\mathrm{Phage}\;\mathrm{amount}\;\mathrm{before}\;\mathrm{burst}}{\mathrm{Amount}\;\mathrm{of}\;\mathrm{infective}\;\mathrm{centers}}$$

where the number of infective centers was considered as same as the number of adsorbed phages (determined by the initially added phage amount minus un-adsorbed phage amount before burst), assuming that one bacterial cell was infected by only one single phage at MOI_added_ of 0.1, based on Poisson distribution [41].

### 2.11. Phage Adsorption Assays

*B. cereus* strain 1582-3B cells at exponential stage (OD_600_ 2.0; 1 × 10^8^ CFU/mL) were harvested (13,000 × *g* at 25 °C for 1 min) and re-suspended in TSB or TSB containing 1 mM CaCl_2_, respectively. The cell suspension was adjusted to an OD_600_ of 0.1 – 0.15 (10^7^ CFU/mL) with the same medium and supplemented with 25 μg/mL of chloramphenicol to inhibit cell growth and phage multiplication [42]. Cell suspension was aliquoted into 2 mL Eppendorf tube and pre-warmed for 10 min at 37 °C together with phage suspension. Then, DLc1 phage was added to each tube to achieve a final concentration of 10^4^ PFU/mL and incubated in a 37 °C water bath. Samples were taken at 5-min intervals until 15 min and filtrated through a 0.22-μm syringe filter. The amount of un-adsorbed free phage particles was determined by the double agar overlay plaque assay.

To evaluate the adsorption efficiency at different temperatures, aliquoted cell suspension above (in TSB with 1 mM CaCl_2_) was pre-warmed together with phage suspension at 4, 25, 30, and 37 °C for 10 min, respectively. After mixing DLc1 with cells and incubating at the corresponding temperature for 15 min, the amount of un-adsorbed phages was determined after filtration as above. A bacteria-free setup served as the control.

To identify the type of bacterial receptors recognized by DLc1, *B. cereus* strain 1582-3B cells were treated with sodium periodate (Shanghai Titan Scientific Co., Ltd., Shanghai, China) or proteinase K (Magen Biotech Co., Ltd., Guangzhou, China) and the adsorption assay was performed as described above. The periodate and proteinase K treatments were carried out following the method described previously [46,47]. Briefly, exponentially growing *B. cereus* strain 1582-3B cells (OD600 2.0; 1 × 10^8^ CFU/mL) were harvested (13,000 × *g* at 37 °C for 1 min) and washed once with TSB. The cell pellets were re-suspended in the following: (1) 1 mM sodium periodate in 50 mM sodium acetate (Aladdin Reagent Co., Ltd., Shanghai, China) (pH 5.2); (2) 10 mM sodium periodate in 50 mM sodium acetate (pH 5.2); (3) 100 mM sodium periodate in 50 mM sodium acetate (pH 5.2); (4) 50 mM sodium acetate (pH 5.2); (5) TSB (the negative control for periodate treatment); (6) 0.2 mg/mL proteinase K in 20 mM Tris-HCl (Beijing Solarbio Science & Technology Co., Ltd., Beijing, China) and 100 mM NaCl (pH 7.5); (7) 20 mM Tris-HCl and 100 mM NaCl (pH 7.5); (8) TSB (the negative control for proteinase K treatment). Samples (1) – (5) were incubated for 2 h at room temperature (28 ± 1 °C) with shaking and protected from light. Samples (6) – (8) were incubated for 2 h at 45 °C. After treatments, the samples were washed three times with TSB and adjusted to an OD_600_ of 0.1 – 0.15 (10^7^ CFU/mL). Adsorption assays were performed as described above using phages in TSB without cells as the blank control.

To evaluate the adsorption efficiency of different strains toward DLc1, the adsorption assay was conducted as above (15 min adsorption at 37 °C) toward selected strains. Three independent experiments were performed and the results were represented as the mean count standard deviations.

### 2.12. Statistics

Statistical analysis was performed using one-way ANOVA and Duncan’s multiple range test in SPSS (version 19, IBM Corporation, Armonk, NY, USA), except for Figure 6E and Figure 9A, which used independent-samples *t*-test for pairwise comparison. The level of significance was set at *p* ≤ 0.05.

### 2.13. Genomic Data Availability

The sequence data for the *Bacillus cereus* phage vB_BceP-DLc1 were deposited at GenBank under the accession number of MW012634.

## 3. Results

### 3.1. Isolation and Morphological Characteristics of DLc1

**Figure 1:**
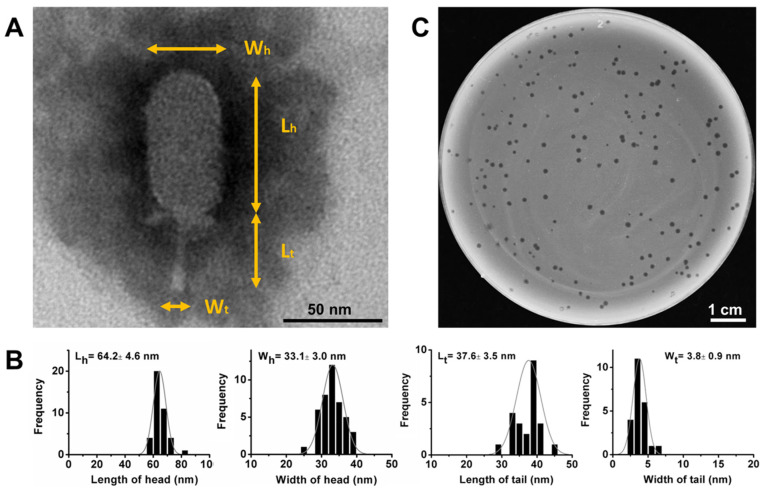
The morphology and size of phage DLc1 and phage plaques. (**A**) TEM of DLc1, indicating each measured part of virions (W_h_, the width of the head; L_h_, the length of the head; W_t_, the width of the tail; L_t_, the length of the tail); (**B**) Average size and statistical histogram of each part measured in at least 20 individual virions; (**C**) Phage plaques formed on a double-layer agar plate (0.4% agar in top layer) using *B. cereus* 1582-3B as the host.

Phage DLc1 was isolated from sewage samples, using *B. cereus* strain 1582-3B as the host, which was isolated from pasteurized milk in Guangzhou, China and found to possess an antimicrobial resistance profile of AMP-AMC-P-KF-FOX-RD and a virulence gene profile of *hblA*-*hblC*-*hblD*-*nheA*-*nheB*-*nheC*-*hlyll*-*entFM*-*bceT* [48]. The phage could form clear plaques of approximately 1-mm diameter in a double-layer TSB agar plate (0.4% agar in top layer) following an incubation period of 4 to 12 h at 37 °C (Figure 1C). The purified and high-titer phage stock was subjected to TEM observation, which revealed that phage DLc1 particle had a head-tail structure containing an elongated head (length 64.2 ± 4.6 nm and width 33.1 ± 3.0 nm) and a short non-contractile tail (length 37.6 ± 3.5 nm and width 3.8 ± 0.9 nm) (Figure 1A,B). The morphology of DLc1 is typical for the order *Caudovirales* and family *Podoviridae*, and the dimensions and tail structure may allow DLc1 to be classified into the *Picovirinae* subfamily [20,49]. Thus, the phage DLc1 was systematically named as vB_BceP-DLc1 according to the proposal of Adriaenssens and Brister in 2017 [50], where “vB” stands for the virus of bacteria, “Bce” for the host organism *B. cereus*, and “P” for the virus family *Podoviridae*.

### 3.2. Genomic and Phylogenetic Analysis

**Figure 2:**
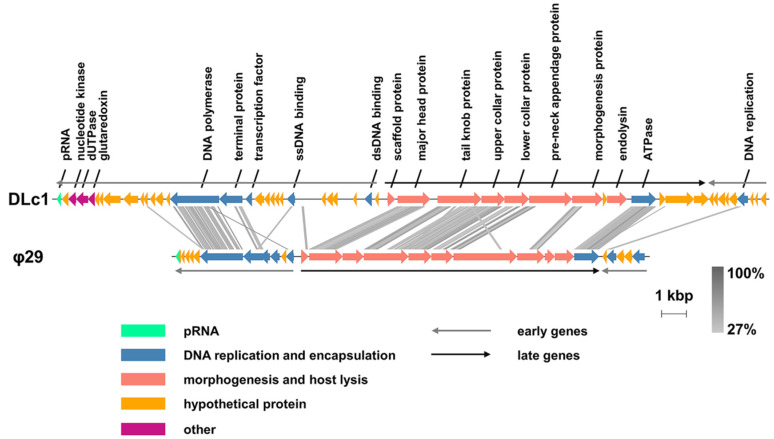
Overview of the DLc1 genome compared to the type strain φ29. Predicted functions of the open reading frames (ORFs) are indicated and the tblastx similarities between two genomes are displayed as vertical grey lines, with grey levels representing the degree of similarity.

Based on the results of next-generation sequencing combined with Sanger sequencing, phage DLc1 showed a linear double-stranded DNA genome with a size of 28,950 bp and GC content of 31.09%, and the genome harbored 5-bp inverted terminal repeats (ITRs) (5′ AAATG-) (Figure S1). There were one non-coding pro-head RNA and 50 putative ORFs predicted for protein-encoding, among which 18 putative ORFs could be assigned with potential functions. No tRNA was found in the genome of DLc1. The predicted functions of different genes in DLc1 are listed in Table S3, which showed a high degree of similarity with phage φ29 in protein composition and arrangement [22], as illustrated in Figure 2. Briefly, identical to the expression patterns of type strain φ29, early genes of phage DLc1 were distributed in the left and right region of the genome and late genes inserted in the middle. A high degree of similarity can be observed in the DNA polymerase and terminal protein in the left early region, which are responsible for the DNA replication, as well as the proteins for morphogenesis and DNA encapsidation in the late region. Meanwhile, the same as φ29 and the φ29-like phages [20], the pRNA of DLc1 was found at the end of left early gene region, which is a crucial part of the DNA-packaging motor specific to the φ29-like phages [51]. As a result, the existence of pRNA, terminal protein, and short ITR region in its genome, together with the distinct morphology, revealed again that the phage DLc1 should be classified into the *Picovirinae* subfamily, maybe especially into the φ29-like phages in the *Salasvirus* genus.

**Figure 3:**
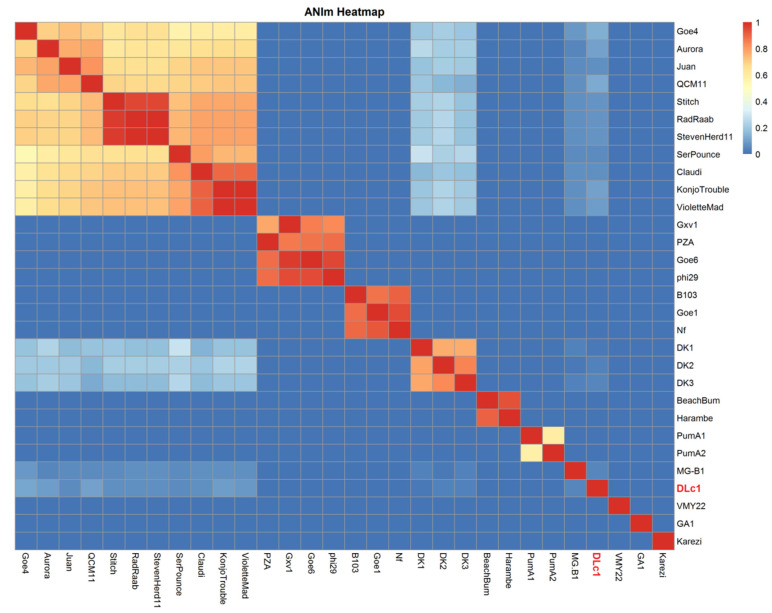
Average nucleotide identity (ANI) analysis of 30 genomes of DLc1 and other φ29-like phages in NCBI. Alignments were performed using the MUMmer (ANIm) method [20] and the Hadamard matrix is presented in a heatmap.

To disclose the relationship of DLc1 to other φ29-like phages, average nucleotide identity (ANI) values were calculated (Table S4). Three typical species in the *Salasvirus* genus (φ29, B103, and GA1) approved in ICTV and another twenty-seven φ29-like phages containing DLc1 were compared pairwise, and the Hadamard matrix was illustrated by a heatmap (Figure 3). The φ29-like phages could be divided into 11 clusters based on the ANIm values. The species φ29, B103, and GA1, and their closely related phages (Goe6, Gxv1, and PZA for φ29 group; Goe1 and Nf for B103 group), were well separated from others, revealing again the existence of three groups in *Salasvirus* genus [22]. The largest cluster contained phage Goe4 and its relatives (Aurora, Juan, QCM11, Stitch, Radaab, StevenHerd11, SerPounce, Claudi, KonjoTrouble, and VioletteMad), consistent with the findings of Schilling et al. [20], except for MG-B1, which was separated as an independent group in our results. The newly isolated phage DLc1 also occupied an independent position, with a certain degree of similarity to the Goe4 cluster (< 11%), MG-B1 (4.5%), and our previously isolated phages DK1, DK2, and DK3 [15] (< 5%). DK1, DK2, and DK3 formed a single cluster as well, with similarity to the Goe4 cluster of 13% to 30%, MG-B1 of 2% to 4%, and DLc1 of 2% to 4%. The remaining phages were split into four clusters including BeachBum and Harambe, PumA1 and PumA2, VMY22, and Karezi, respectively.

**Figure 4:**
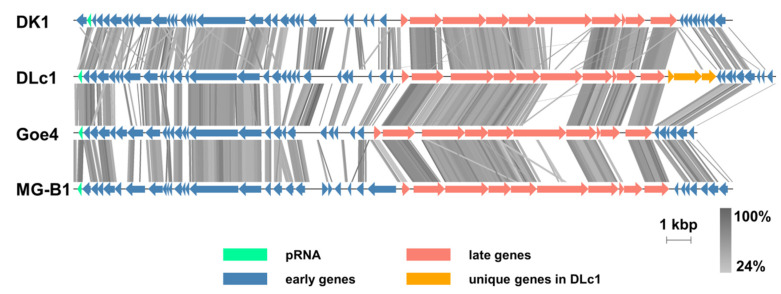
Pairwise genome alignment of DLc1 with its most related phages. The tblastx similarities are displayed as vertical grey lines, with grey levels representing the degree of similarity. The unique inserted genes in DLc1 are indicated as yellow arrows.

The pairwise genome alignment of DLc1 with DK1, Goe4, and MG-B1, which are representatives of the related groups to DLc1 presented in ANI analysis (Figure 3), is displayed in Figure 4. All viruses showed consistent genome organization, with pRNA at the end of the left early gene region, except for DK1, in which pRNA is the penultimate. Similar to the results of Schilling et al. [20], a non-coding genomic region between the genes encoding the major head protein and the tail knob protein was found in both Goe4 and DLc1, but absent in DK1 and MG-B1. High diversity can be found in the genes encoding pre-neck appendage protein among these phages, which has been proven to be crucial in the recognition of the hosts by the phages [52]. It is worth noting that a three-gene set in the late region was observed between the genes encoding DNA encapsidation ATPase and the right early region, which is one of the major contributors to the largest genome of DLc1 (Table S1). Interestingly, this region is absent in all other φ29-like phages [20].

**Figure 5:**
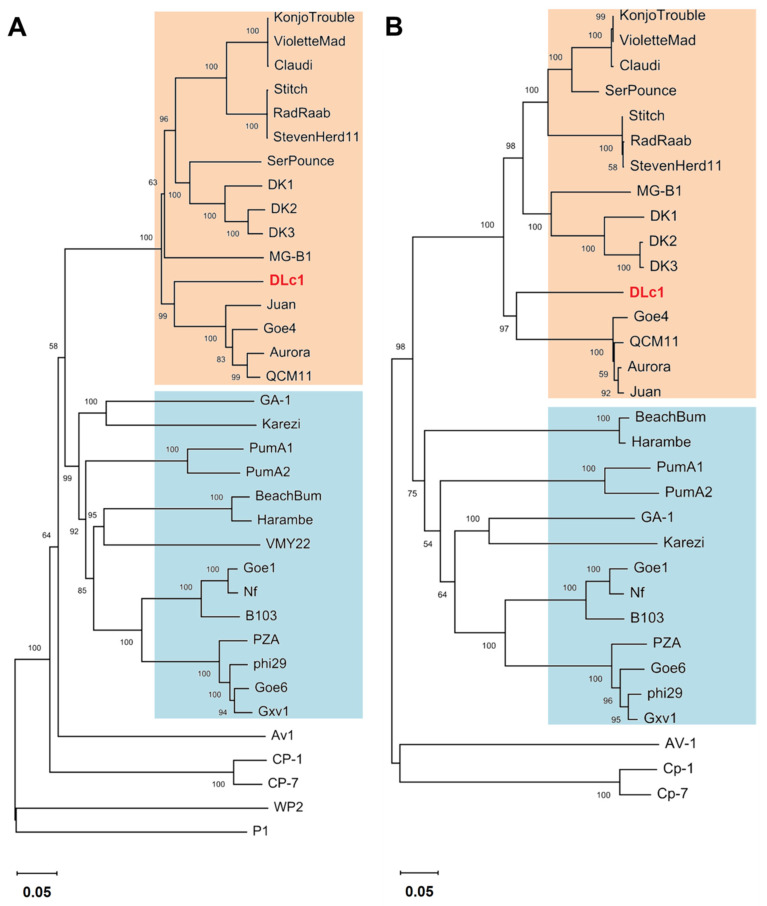
Phylogenetic classification of the φ29-like phages and other members sorted in the current *Picovirinae* subfamily. The genes encoding DNA polymerase (**A**) and terminal protein (**B**) are set as markers to construct the phylogenetic tree, respectively. Two clusters are colored in orange or blue background, respectively.

The phylogenetic relationship between DLc1, other φ29-like phages, and five members of the *Picovirinae* subfamily is shown in Figure 5. The genes for DNA replication (DNA polymerase and terminal protein), for host recognition (pre-neck appendage protein), and for progeny releasing (endolysin) were selected as the markers. The DNA polymerase was the only conserved protein in all members of the *Picovirinae* subfamily, and the phylogenetic tree generated by genes coding DNA polymerase and terminal protein revealed a similar grouping result as shown in ANI analysis (Figure 3). The phages DLc1, MG-B1, and DK1 are more similar to the Goe4 group regarding the DNA polymerase and terminal protein sequences, indicating an evolutionary difference among these phages to other φ29-like phages. In addition, the phylogenetic clusters in the pre-neck appendage protein and endolysin were more inconsistent with the previous grouping results (Figure S2), suggesting that the proteins involved in host recognition and progeny releasing may not be suitable for phylogenetic analysis [20].

### 3.3. Stability of DLc1 to Environmental Stresses

**Figure 6:**
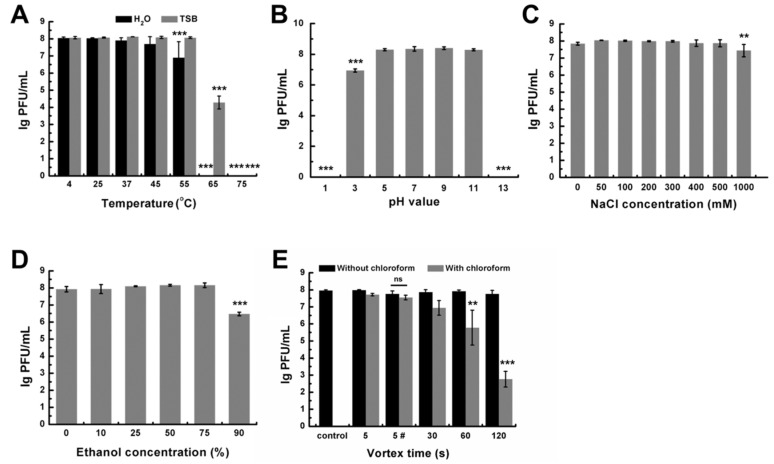
Stability of DLc1 under different environmental stresses, including (**A**) temperature (statistical significance is done by comparing the value of each temperature with that of 4 °C); (**B**) pH (statistical significance is done by comparing the value of each pH with that of pH 7); (**C**) NaCl concentration (statistical significance is done by comparing the value of each concentration with that of 0 mM); (**D**) ethanol concentration (statistical significance is done by comparing the value of each concentration with that of 0%); (**E**) mechanical vortex and the existence of chloroform. 5 # represents the vortex time of 5 s followed by sitting at 4 °C for 24 h (statistical significance in mechanical vortex without chloroform is done by comparing the value of different vortex time to that of control, and in the condition with chloroform, statistical significance is done by comparing the value of different vortex time to that of 5 s; statistical significance is also compared between values with and without chloroform in 5 #). Means and standard deviations (SD) of three independent assays are shown. Asterisks indicate significant differences (** *p* ≤ 0.01 and *** *p* ≤ 0.001), and ns represents no significant difference.

Infectivity of DLc1 under different environmental stresses was determined. When incubated at different temperatures for 1 h in TSB, phage DLc1 exhibited a high degree of stability at the temperature ranging from 4 to 55 °C, and the activity dramatically declined from 65 °C and was entirely lost at 75 °C (Figure 6A). Moreover, DLc1 showed worse thermal tolerance in ultrapure water. The infectivity decreased significantly from 55 °C and was completely lost at 65 °C (Figure 6A). DLc1 displayed constant activity over a range of pH values from 5 to 11 (Figure 6B) and in the presence of NaCl up to 500 mM (Figure 6C). The titer decreased slightly in 1 M of NaCl. Additionally, DLc1 could resist the treatment of aqueous ethanol at concentrations up to 75% (Figure 6D), which is the commonly used for disinfecting microorganism [53]. The phage DLc1 showed chloroform resistance in the conditions of a transient vortex (5 s) or static storage, as shown in Figure 6E, but nearly lost its activity after vortex for longer periods (>30 s) together with chloroform. In the absence of chloroform, phage DLc1 exhibited good mechanical stability toward vortex (Figure 6E).

### 3.4. Host Range of DLc1

**Figure 7:**
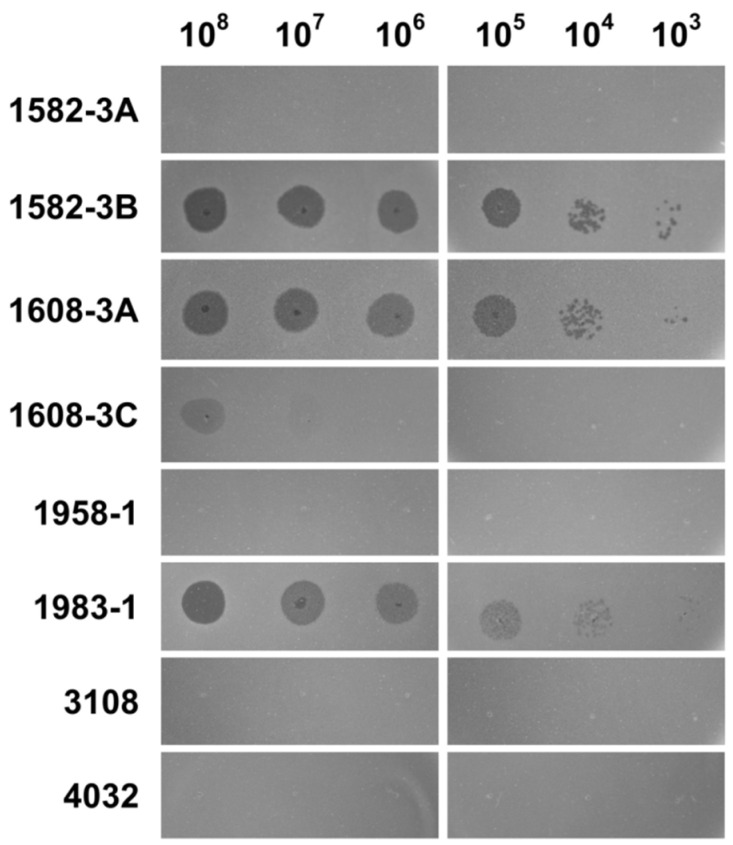
The spot assay on ST4 *B. cereus* strains by DLc1 with stepwise dilutions. Four strains (1582-3B, 1608-3A, 1608-3C, and 1983-1) show sensitive response, and other four strains (1582-3A, 1958-1, 3108, and 4032) present insensitive response.

To determine the potential host range of the phage DLc1, the spot test assay was performed against 77 strains of *B. cereus* isolated from pasteurized milk in China [48] together with a type strain ATCC 14579. As shown in Table 1, four out of 78 strains were sensitive to DLc1, and all the sensitive strains belonged to the sequence type (ST) 4, which revealed the narrow host range and high host-specificity of DLc1. Nevertheless, in the four sensitive strains, diluted DLc1 resulted in individual plaques on *B. cereus* strain 1582-3B, 1608-3A, and 1983-1, but only less clear plaque on *B. cereus* 1608-3C (Figure 7).

### 3.5. Adsorption Rate Constant and One-Step Growth Curves

**Figure 8:**
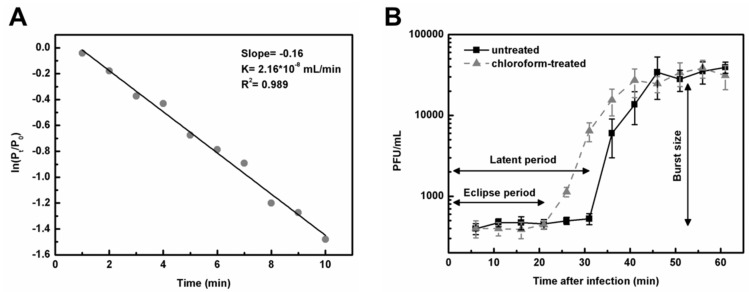
Adsorption rate constant and one-step growth curve of DLc1 using *B. cereus* 1582-3B as the host. (**A**) The representative linear fitting result of a single adsorption rate constant determination at a MOI_added_ of 0.001; (**B**) One-step growth curve of DLc1 at a MOI_added_ of 0.1. The experimental conditions were both set at 37 °C in TSB supplemented with 1 mM CaCl_2_.

Under the same conditions, multiple experimental repeats with single sampling per time point were conducted to minimize the error in *k* value estimation [41], and the representative linear fitting results of a single experiment are shown in Figure 8A. The results for other repeats can be found in Figure S3 as well. The adsorption rate constant of DLc1 to its host *B. cereus* 1582-3B was 2.08 to 2.38 × 10^-8^ mL/min, with a good fitting degree (R^2^ = 0.989). The life cycle and infective capacity of DLc1 toward its host *B. cereus* 1582-3B were further examined in a one-step growth curve (Figure 8B). The eclipse period and the latent period were around 21 and 31 min, respectively. The average burst size was 20 phage particles per infected cell.

### 3.6. Phage Adsorption Assay

**Figure 9:**
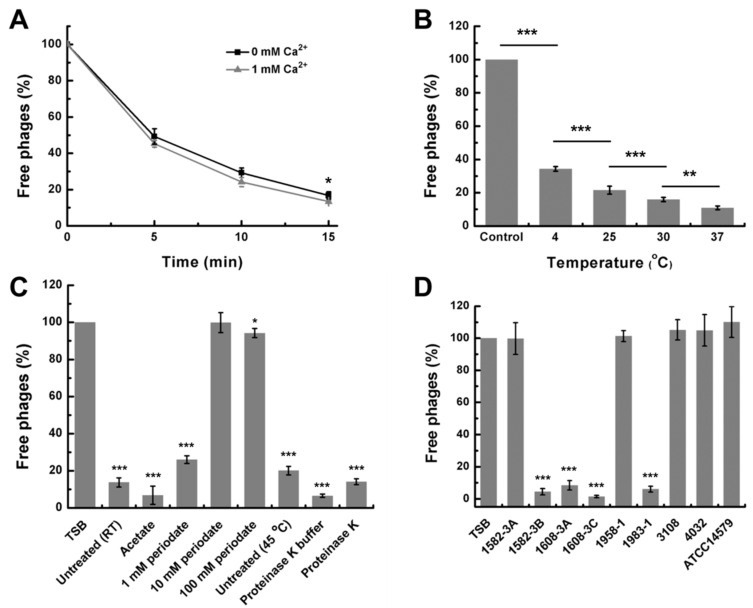
Adsorption assay of DLc1. (**A**) Amount of adsorption during 15 min using 1582-3B as the host at 37 °C; (**B**) Adsorption of DLc1 to 1582-3B at different temperatures for 15 min; (**C**) Adsorption of DLc1 to 1582-3B after different treatments on the host cells (statistical significance is done by comparing the value of each group with the TSB group); (**D**) Adsorption of DLc1 to different strains (statistical significance is done by comparing the value of each group with the TSB group). (**C**) and (**D**) were both conducted at 37 °C for 15 min. All data present the means and standard deviations (SD) of three independent assays. Asterisks indicate significant differences (* *p* ≤ 0.05, ** *p* ≤ 0.01, and *** *p* ≤ 0.001).

The adsorption curve of DLc1 toward *B. cereus* 1582-3B is plotted in Figure 9A. The majority of DLc1 particles (about 85%) could attach to the bacteria within 15 min at 37 °C in TSB, with or without 1 mM CaCl_2_. As a result, the subsequent adsorption assays were conducted within 15 min under the same conditions with 1 mM CaCl_2_. As shown in Figure 9B, the amount of adsorbed DLc1 on *B. cereus* 1582-3B decreased with the decrease in temperature, demonstrating a temperature-dependent adsorbing capacity.

To test which kinds of receptors on the cell surface can be recognized by DLc1, *B. cereus* 1582-3B was pre-treated with periodate, which can cleave the carbohydrates structures with vicinal diols [46], or proteinase K, which can destroy the proteins located on the surface, before the adsorption assay. The host cells treated by periodate at concentrations of 10 and 100 mM could significantly inhibit the adsorption of DLc1, whereas the treatment by proteinase K did not yield any obvious effect (Figure 9C). Therefore, DLc1 seemed to use carbohydrates but not proteins as the receptor to adsorb. Similar results were obtained from the TEM observation, in which a mass of phages bind to the surface of the host cell without treatment (Figure S4A,B); however, only a few phages could adsorb to the cells treated with periodate (Figure S4C). On the other hand, the proteinase K-treated cells seemed to be surrounded by a loose layer of carbohydrates, which were still bound by a large number of phages (Figure S4D).

Considering that DLc1 only infects strains belonging to ST4 (Table 1), all eight strains of this type were further tested by phage adsorption. As shown in Figure 9D, DLc1 could largely adsorb onto different sensitive strains 1582-3B, 1608-3A, 1608-3C, and 1983-1; however, the adsorption did not occur on other strains with the same ST.

## 4. Discussion

### 4.1. Classification Proposal

As recommended by ICTV [54], a crude estimate of the overall and nucleotide identity could be obtained by multiplying the Blastn query coverage by identity. Therefore, the newly isolated *B. cereus* phage DLc1 achieved a maximal nucleotide identity of just 22.5% toward its closest phages in NCBI (Table S5). Following the current demarcation criteria for bacterial and archaeal viruses described by the Bacterial and Archaeal Viruses Subcommittee (BAVS) of ICTV [50], the main species demarcation criterion is less than 95% identity of the genome sequence, and for species in the same genus, it is > 50% similarity of the nucleotide sequence. Thus, DLc1 is much different from all existing species.

To obtain more distinct references in the taxonomy of DLc1, holistic approaches, relying on the morphology, genome synteny, average nucleotide identity, and phylogenetic analysis, were employed. From its special morphology and a high degree of genome synteny with phage φ29, DLc1 can be considered as a new member of the φ29-like group, sharing a common protein-primed DNA replication mechanism, pRNA-facilitated DNA packaging, and homogeneous genes encoding proteins for morphogenesis and host lysis in the late gene region. Until recently, only a fraction of these viruses has been classified into the *Picovirinae* subfamily of the *Podoviridae* family, including B103, GA1, and the well-known type strain φ29 [24], which are integrated into genus *Salasvirus* together with an insufficiently informed strain *Kurthia* virus 6. These species represent three groups of the φ29-like phages historically [22] and are well separated into three clusters in the current ANI analysis together with their closely related phages (Figure 3). ANI analysis has been proposed to be one of the best approaches to determine species boundary and confirm the identity [55]. In this study, we constructed a Hadamard matrix [56], which represented the product of the ANI and the percent of the genome aligned between every pairwise comparison of 30 completed genomes from the φ29-like phage group. Among these unclassified φ29-like phages, increasing diversity was presented, consistent with the results stated before [20]. Besides the diversity in *B. subtilis* phages (φ29, B103, and GA1 clusters) and *B. thuringiensis* phages (Goe4, Harambe, and Karezi clusters), recently available *B. cereus* phages were also split into different clusters (DK1, VMY22, and DLc1). According to the recommendation by ICTV, each of these clusters sharing < 50% similarity of the nucleotide sequence could be regarded as a separate genus. Nevertheless, the position of a new virus in taxonomy should also be comprehensively considered by combining the host of the virus, its genome relativeness, protein homologs, and phylogeny. Based on their common morphology, genomic organization, and host species (*Bacillus* species), the φ29-like phages including the newly isolated DLc1 are currently proposed to categorize into the existing genus *Salasvirus*. However, as displayed by the phylogenetic analysis (Figure 5), the phage DLc1 located in the cluster containing the Goe4 group, DK1 group, and MG-B1 (i.e., Goe4 cluster) that is distinctly separated from those including *B. subtilis* phages, and some other *B. thuringiensis* (Harambe group and Karezi) and *B. cereus* (VMY22) phages. In addition, all members of the Goe4 cluster possess larger genomes (> 20 kb) and less G + C % content (30% – 32%) (Table S1). Therefore, we proposed that the existing genus *Salasvirus* should be expanded to all of the φ29-like phages considering the above similarities and differences. Meanwhile, subgenus could be established to preferably separate phages with different features, e.g., subgenus containing the Goe4 cluster or the φ29 cluster.

### 4.2. Genomic Features

It is noteworthy that DLc1 has the largest genome at present among all φ29-like phages (1744 bp larger than the second one, SerPounce), which is partially due to the insertion of a three-gene cluster (ORF 39, ORF 40, and ORF 41) at the right end of its late gene region (Figure 4). The inserted gene cluster is unique for DLc1 and could not be found in other φ29-like phages (see Figure 4 and [20]). It was predicted by the NCBI tblastx and the InterProScan that the ORFs 40 and 41 are responsible for encoding DNA translocase FtsK and replication relaxation family protein, respectively, and the product of ORF 39 contains transmembrane domains. This three-gene insertion in DLc1 may be originated from the bacterium [57], implying the participation of bacteriophages in the horizontal gene transfer (HGT) [58].

Another major difference among the aligned phages is the gene encoding the pre-neck appendage protein, which has been proven to take part in the recognition and adsorption of the hosts by the phages [52]. Due to the different species of their host, i.e., *B. thuringiensis* (Goe4), *B. weihenstephanensis* (MG-B1), and *B. cereus* (DK1 and DLc1), it can be speculated that the receptors of these phages are also distinct [20]. Even within the same species, for example, the receptors can be different between different strains of *B. cereus*, making the diversity take place at the genetic level (Figure 4).

### 4.3. Interaction between DLc1 and Host

Once phages are encountered with their host cells, irreversible adsorption occurs if phage receptors are available. Roughly, there are two kinds of receptors located on the cell wall of Gram-positive bacteria, including proteins and polysaccharides [59]. Considering the phages infecting *Bacillus* species, both receptors are possible. It has been reported that phage φ29 can utilize D-glucose in teichoic acid as the receptor [60], whilst the membrane protein encoded by *yueB* can also be used as the receptor for the adsorption of phage SPP1 [61]. In this study, phage DLc1 was not able to adsorb on the surface of *B. cereus* 1582-3B treated by periodate (Figure 9C), which destroyed the structures of carbohydrates such as teichoic acid [46]. We speculate that DLc1 also uses teichoic acid as the receptor because the appendages of the φ29-like phages have been supposed to harbor an enzymatic activity that hydrolyzes the teichoic acid to pull the phage closer to its host [52]. Thus, the receptor of DLc1 is probable a carbohydrate structure on the cell wall and the exact identity needs to be further investigated in the future.

The host range of DLc1 was relatively narrow, restricted to the most related *B. cereus* strains of the same ST. It can be therefore implied that these sensitive strains with the same housekeeping gene sequence may also harbor identical receptors for DLc1. However, DLc1 cannot form plaques to some other tested strains belonging to ST4 in the spot test, i.e., *B. cereus* strain 1582-3A, 1958-1, 3108, and 4032 (Figure 7). Besides this, these insensitive strains cannot be adsorbed by DLc1, as demonstrated in the adsorption assay (Figure 9D). Interestingly, in the four sensitive strains, only 1608-3C showed blurry plaques when spotted with less diluted DLc1 suspension, and the plaques disappeared in high dilutions (Figure 7). Since *B. cereus* 1608-3C can be also largely adsorbed by DLc1, as with other sensitive strains (Figure 9D), the resistance of this strain to DLc1 could be due to some other reasons, such as the CRISPR-Cas defense system [62], rather than the adsorption inhibition. The occurrence of blurry plaques in 1608-3C under high-titer phage spotting and the absence of individual plaques under higher dilutions could be the situation of “lysis from without” [63], in which the bacterial lysis is induced by phage adsorption at high multiplicity and no progeny is produced. The detailed phage-tolerance mechanism of this strain needs to be further studied and this new phage should be a promising object to explore the virus–host coevolution relationship.

### 4.4. The Application Potential

As a lytic phage, DLc1 could be capable of controlling *B. cereus*. The thermo-stability of DLc1 is fairly high, especially in TSB, and relatively lower in pure water (Figure 6A) since a medium lacking ionic environment would be disadvantageous for maintaining the structure stability of the proteins at higher temperatures [64]. Nevertheless, the phage could keep an invariably high titer at least for 1 year when stored at 4 °C in pure water. Additionally, DLc1 also showed favorable pH- and salt-tolerance, making it more preferable as a bio-controlling agent in the food industry, where various conditions could be encountered during food processing and preservation [65]. When compared with some other *B. cereus* phages [66,67,68], the burst size of DLc1 is relatively low and the latent period is shorter (Figure 8B). Although it has been shown that a large burst size is preferred in phage therapy [69], DLc1 with a short latent period and high adsorption efficacy also displayed its advantages.

DLc1 also exhibits good stability in high concentrations of ethanol solutions, and 90% (v/v) ethanol solution might lead to some degree of dehydration in proteins and influence its activities [70]. The phage was also very stable under the treatment of chloroform. It has been reported that the treatment of chloroform will decrease the infectivity of many phages, especially for those possessing head-fibers, which probably participate in the infection [71]. As displayed in morphology and genome orientation (Figure 1A and Figure 2), DLc1 did not possess lipid fibers on its capsid, possibly leading to its stability. The stability of DLc1 in different solvents is advantageous for its application as nanoparticles in biomedical fields, where phage particles have attracted lots of attention owing to their monodispersed and rigid construction [72]. Moreover, DLc1 displayed extraordinary stability under vigorous mechanical disturbance (vortex), possibly due to the excellent stiffness of its capsid, as demonstrated in phage φ29 [73]. The declined infectivity under shaking in the existence of chloroform could be attributed to the disruption of hydrophobic interactions in amino acids, resulting in the destabilization of proteins [74,75].

## 5. Conclusions

In this study, the largest member of φ29-like phages, phage vB_BceP-DLc1, at present has been identified. The phage DLc1 has a unique inserted gene cluster, contributing its large genome. Based on the morphology, genome synteny, average nucleotide identity, and phylogenetic analysis, the phage DLc1 can be categorized into the existing genus of *Salasvirus*. Meanwhile, a new classification of all the φ29-like phages was proposed, considering the currently incomplete collection in ICTV. Dlc1 can use surface carbohydrate structures of the host cell as the receptors and can only infect the most related *B. cereus* strains. Although the burst size of DLc1 is relatively low, the short latent period and high adsorption efficacy of DLc1 are advantageous for its application in phage therapy. DLc1 showed high stability under different conditions, which makes this new phage a potential candidate as an antimicrobial agent or nano-carrier in the food and pharmaceutical industries.

## Figures and Tables

**Table 1:** Sensitivities of different *B. cereus* strains to phage DLc1.

No.	Strain Name	ST	Sensitivity ^a^	No.	Strain Name	ST	Sensitivity
1	ATCC14579	921	−	40	3858-1B	205	−
2	1582-3A	4	−	41	3958	205	−
3	1582-3B	4	+	42	3982-3A	205	−
4	1608-3A	4	+	43	4182-3C	217	−
5	1608-3C	4	+	44	2233-1	371	−
6	1958-1	4	−	45	2233-2	371	−
7	1983-1	4	+	46	2432-2	462	−
8	3108	4	−	47	3482-2C	512	−
9	4032	4	−	48	3483	512	−
10	233-1	18	−	49	82	770	−
11	233-2	18	−	50	108-1	770	−
12	3332	24	−	51	208	770	−
13	3332-1A	24	−	52	1058-2	770	−
14	3332-2A	24	−	53	1682-2B	770	−
15	791	26	−	54	2008-2	770	−
16	1058-1	26	−	55	3308-1A	770	−
17	2132-4	26	−	56	3208	869	−
18	2583	26	−	57	3233	869	−
19	2932	26	−	58	2083-2	938	−
20	4182-1C	26	−	59	2008-3	962	−
21	1782-2B	32	−	60	2208	1001	−
22	2083-1	59	−	61	3808	1032	−
23	58	90	−	62	276-1C	1084	−
24	232	90	−	63	Y608	1225	−
25	1833-1C	90	−	64	3008-1B	1237	−
26	2833	90	−	65	3732	1327	−
27	3532	90	−	66	2833-2A	1328	−
28	1682-3B	92	−	67	4233	1329	−
29	1782-3A	92	−	68	4233-1A	1329	−
30	Y1808	92	−	69	892-1	1330	−
31	2042-2	104	−	70	3283	1332	−
32	3232	144	−	71	1958-2	1418	−
33	3258-2A	144	−	72	2132-1	1419	−
34	1682-2C	158	−	73	1782-1A	1435	−
35	Y1683	164	−	74	3132-3A	1439	−
36	1782-3B	164	−	75	2833-1A	1440	−
37	Y641	172	−	76	2833-3A	1440	−
38	1127	184	−	77	1558-2A	1565	−
39	183	205	−	78	1558-3B	1565	−

ST, sequence type; ^a^ +, sensitive (plaque formed); −, insensitive (no plaque formed).

## Supplementary Materials

The following are available online at https://www.mdpi.com/2076-2607/8/11/1750/s1, Figure S1: Sanger sequencing chromatogram of the genome ends of DLc1 verified the 5 bp inverted terminal repeats (5′ AAATG in left end (A) and 3′ TTTAC in right end (B); Figure S2: Phylogenetic tree of the φ29-like phages and other members in the current *Picovirinae* subfamily, constructed with the genes encoding pre-neck appendage protein (A) and endolysin (B), respectively; Figure S3: The linear fitting results of three independent adsorption rate constant determinations; Figure S4: Imaging the adsorption of DLc1 onto *B. cereus* 1582-3B at an MOI_added_ of 100 after different treatments by TEM; Table S1: The viruses used for genome alignment; Table S2: The predicted position of pro-head RNA; Table S3: The predicted functions of ORFs in DLc1 and the similarities of different ORFs to those of phage φ29; Table S4: The Hadamard matrix derived from the ANIm and ANIb alignment method; Table S5: The blastn results in NCBI and the nucleotide identity of DLc1 to its relatives.
