# Supplementary material for: Isolation and Characterization of Bacillus cereus Phage vB_BceP-DLc1 Reveals the Largest Member of the Φ29-Like Phages
Source: Microorganisms. 2020 Nov 7;8(11):1750. doi: 10.3390/microorganisms8111750 (PMC7695010; doi:10.3390/microorganisms8111750)
Supplement: Supplementary file 1 [file microorganisms-08-01750-s001.zip › microorganisms-978159-si-revised/microorganisms-978159-sl.docx]

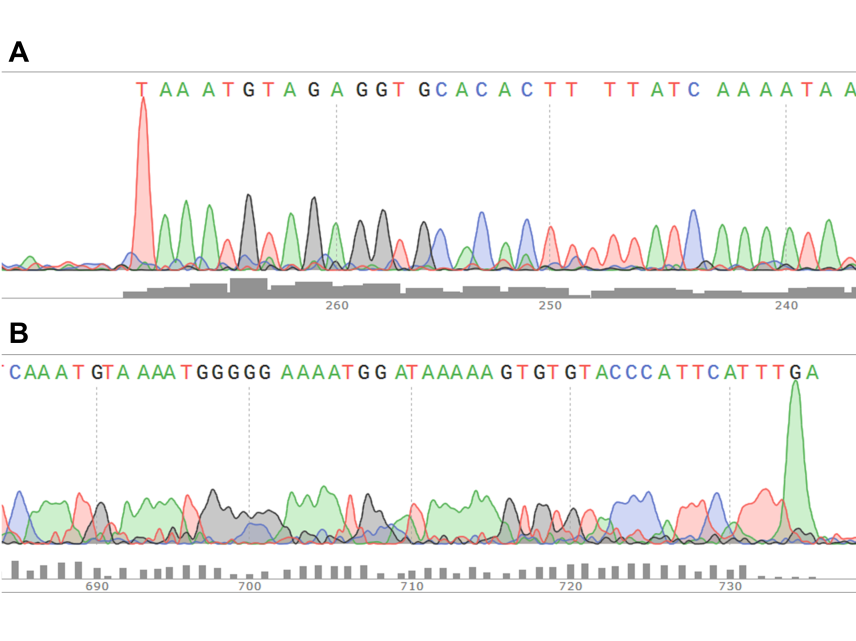


**Figure S1.** Sanger sequencing chromatogram of the genome ends of DLc1 verified the 5 bp inverted terminal repeats (5’ AAATG in left end (**A**) and 3’ TTTAC in right end (**B**). The end of sequence is indicated by an artificial added glorious adenine peak. The figure is visualized through SnapGene (version 2.3.2) and (**A**) has been reverse complemented for clarity.


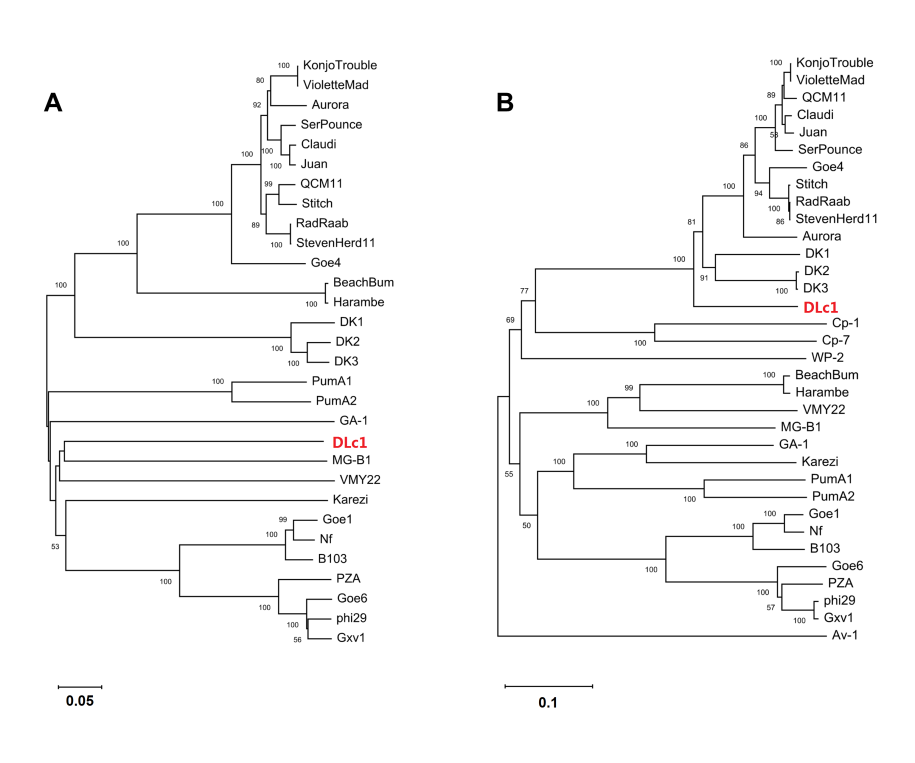


**Figure S2.** Phylogenetic tree of the φ29-like phages and other members in the current *Picovirinae* subfamily, constructed with the genes encoding pre-neck appendage protein (**A**) and endolysin (**B**), respectively.





**Figure S3.** The linear fitting results of three independent adsorption rate constant determinations. The adsorption conditions were set at 37 °C in TSB supplemented with 1 mM CaCl_2_, at an MOI_added_ of 0.001.


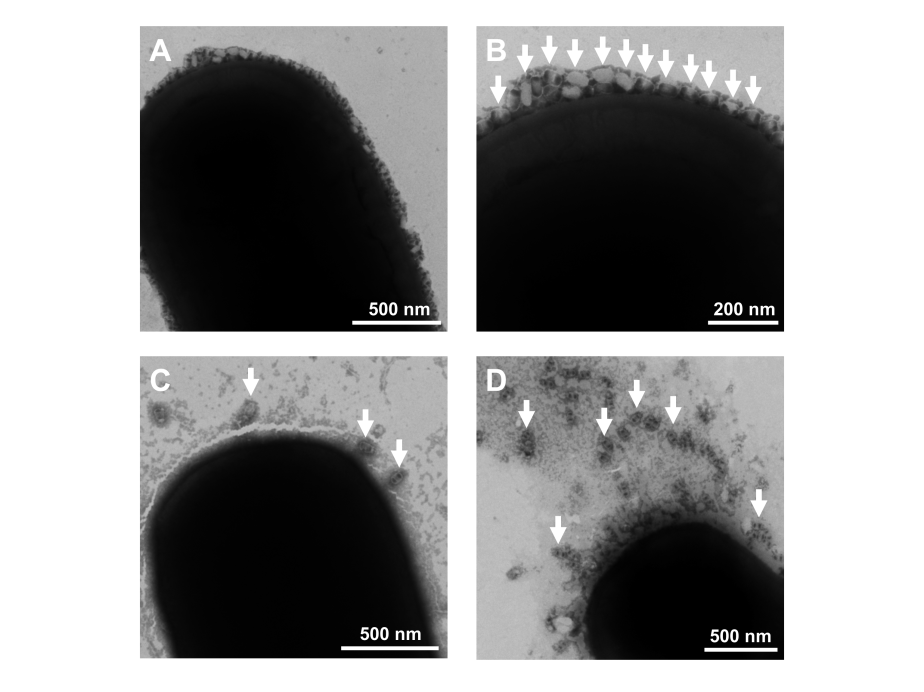


**Figure S4.** Imaging the adsorption of DLc1 onto *B. cereus* 1582-3B at an MOI_added_ of 100 after different treatments by TEM. (**A**) Without treatment; (**B**) Enlargement of (A); (**C**) Treated with 10 mM sodium periodate; (**D**) Treated with proteinase K. All treatments were conducted as described in *in vitro* adsorption assays, and the adsorption time was 10 min at 37 °C. The locations of phages are indicated by white arrows.
